# Generating Explainable and Effective Data Descriptors Using Relational Learning: Application to Cancer Biology

**DOI:** 10.1007/978-3-030-61527-7_25

**Published:** 2020-09-19

**Authors:** Oghenejokpeme I. Orhobor, Joseph French, Larisa N. Soldatova, Ross D. King

**Affiliations:** 8 University of Bari Aldo Moro, Bari, Italy; 9 Aristotle University of Thessaloniki, Thessaloniki, Greece; 10 Open University of Cyprus, Nicosia, Cyprus; 11 Dalhousie University, Halifax, NS Canada; 12 Department of Chemical Engineering and Biotechnology, University of Cambridge, Cambridge, CB3 0AS UK; 13 Department of Computing, Goldsmiths, University of London, London, SE14 6AD UK; 14 The Alan Turing Institute, London, NW1 2DB UK; 15 Department of Biology and Biological Engineering, Chalmers University of Technology, 412 96 Gothenburg, Sweden; 16 Manchester, UK

**Keywords:** Relational learning, Inductive logic programming, Gene expression

## Abstract

The key to success in machine learning is the use of effective data representations. The success of deep neural networks (DNNs) is based on their ability to utilize multiple neural network layers, and big data, to learn how to convert simple input representations into richer internal representations that are effective for learning. However, these internal representations are sub-symbolic and difficult to explain. In many scientific problems explainable models are required, and the input data is semantically complex and unsuitable for DNNs. This is true in the fundamental problem of understanding the mechanism of cancer drugs, which requires complex background knowledge about the functions of genes/proteins, their cells, and the molecular structure of the drugs. This background knowledge cannot be compactly expressed propositionally, and requires at least the expressive power of Datalog. Here we demonstrate the use of relational learning to generate new data descriptors in such semantically complex background knowledge. These new descriptors are effective: adding them to standard propositional learning methods significantly improves prediction accuracy. They are also explainable, and add to our understanding of cancer. Our approach can readily be expanded to include other complex forms of background knowledge, and combines the generality of relational learning with the efficiency of standard propositional learning.

## Introduction

Effective data representations are the key to success in machine learning (ML) [28]. Most ML is based on data representations that use tuples of descriptors, i.e. the data can be put into a single table, where the descriptors (attributes) are the columns, and the examples are rows. Descriptors are properties of the examples that are believed to be important: for example if one wishes to classify pictures of animals then image pixel values are useful descriptors. Such tuple-based representations are essentially based on propositional logic [24]. The effectiveness of the propositional descriptors used for learning can vary greatly, and traditionally, most of the effort in ML went into hand-crafting effective descriptors. This has changed with the success of deep neural-networks (DNNs), which has been based on their capacity to utilize multiple neural network layers, and large amounts of data, to learn how to convert raw propositional descriptors (e.g., image pixel values) into richer internal representations that are effective for learning. Thanks to this ability DNNs have succeeded in domains that had previously proved recalcitrant to ML, such as face recognition and learning to play Go. The archetypal success is face recognition, which was once considered to be intractable, but can now be solved with super-human ability on certain limited problems [20]. *Therefore, a key lesson of the success of DNNs is: use ML to learn better data representations for ML*.

For many problems the standard propositional representation of data is problematic, as such a representation cannot efficiently express all the known relational structure (background knowledge) in the data. In some cases this structure can be encoded for using special purpose methods. For example convolutional neural networks encode relational information about the position of descriptors in the structure of the net. Similarly, recurrent neural networks encode information about temporal structure in the net, graph neural networks encode graphical information, etc. In many cases such special purpose methods can work very well. However, these methods must be redesigned for each new type of problem, and the structure encoded in the learning process is not explicit. It would be more beneficial (and elegant) if the learning biases in DNNs were explicit, and not inherent in the structure of the network. A more general approach to encoding known structure in data is to use logic programs [21] to represent the data – relational learning (RL) [24]. Such programs can express spatial, temporal, graphical structure, etc. using a single formalism, and, crucially, this structure is explicit instead of being implicit (e.g. in the connection of neurons). Logic programs provide a unified way of representing the relations between objects. They also promote explainable ML, as it is usually straightforward to translate logic programs into a series of easily understandable sentences that can be interpreted by domain experts. More formally, logic programs are a subset of 1st-order predicate logic, and therefore more general that propositional representations.

The main disadvantages of using a relational representation compared to a standard propositional one are that RL is more computationally expensive and difficult, as the search space of possible models is much larger, and that RL technology is much less developed. This suggests a hybrid strategy where RL is used to learn effective descriptors, and then standard ML is used to learn the final model [6]. This hybrid approach is particularly suited to problems where the data is semantically complicated, and where symbolic explainable models are required. In such problems RL has the potential to effectively learn new descriptors that are understandable to domain experts. Many biomedical ML problems are potentially suitable for a hybrid RL approach, such as understanding the mechanism of cancer drugs. In this problem one needs to encode background knowledge (problem structure) about gene/protein function, associated pathways, known drug targets, cancer cell type, the molecular structure of drugs, etc. We took data on this problem from the Library of Integrated Network-based Cellular Signatures [18] (LINCS). Specifically, we used the Phase II data, which consists of gene expression levels for 978 landmark human genes under perturbation conditions, making this a regression problem. The perturbation conditions consist of a cancer drug added to a cancer cell line, and it is worth noting here that only the response gene expression values are provided, and one would need to independently construct the input variables from the provided metadata.

We hypothesized that we could improve both ML model explainability, and predictive accuracy, by including additional background knowledge in the learning process using a hybrid RL approach. A key source of this background knowledge was the Stanford Biomedical Network Dataset Collection [22] (SBND). Using RL we mined frequent patterns about each drug found in relation to additional background knowledge. These patterns are expressed in Datalog [8] and are explainable to domain experts. They can also be used as binary descriptors in standard ML methods. It is worth noting that ML model explainability heavily depends on the learning algorithm, as some learning algorithms are more interpretable than others. However, we argue that the descriptors generated using the hybrid RL approach will generally be more interpretable than their propositional counterparts.

We evaluated the predictive performance of the newly learnt RL descriptors versus the standard descriptors, both when used by standard ML in isolation, and in combination. We compared two approaches to combining sets of descriptors: one in which the features from both representations are concatenated to form a single dataset, and another where predictions are stacked [2]. We found that the standard descriptors generally outperform the RL descriptors when used in isolation. However, the RL descriptors significantly improve predictive performance when used in combination. Moreover, these new effective RL descriptors are understandable by domain experts. The main contributions of the paper are as follows:

1. Demonstration of the effectiveness of hybrid RL learning on an important real-world problem.
2. Learnt explainable patterns underlying common cancer drugs.
3. A fully integrated biomedical knowledge base in Datalog.

## Related Work

The problem of building models to predict drug effects has been widely studied, from potential adverse effects [33] and drug-drug interactions [32] to cancer cell sensitivity [23]. One such task is the learning of quantitative structure activity relationships (QSARs), where one is interested in predicting the effect of a drug or chemical compound from its molecular structure [25]. Molecular structure is usually represented using molecular fingerprints, which are tuples of Boolean descriptors [5]. However, several other approaches also exist. For example, some authors have used the 3-dimensional structure of chemicals [35], while others have extracted molecular vector embeddings using graph neural networks [15]. In our evaluation, we used the most widely adopted molecular fingerprint representation as the propositional approach. The LINCS data has been used in several studies, e.g. for the task of predicting gene expression levels using perturbation conditions [4]. In contrast to our evaluation, the authors do not utilise background knowledge in the learning process.

Several techniques have been applied to interconnected knowledge bases for various problems in biology [1, 37]. RL in particular has been used in problems such as predicting gene function [16], gene regulation [9] and QSAR-related problems [31]. RL algorithms such as WARMR, which we use in our evaluation, have been shown to be successful in identifying relationships in linked data [17]. However, there are other algorithms like AMIE [10] which have also been shown to perform remarkably well. Furthermore, there exist several other approaches for learning representations from graph or inherently relational data [3, 13] with varying levels of predictive performance and interpretability. We argue that our decision to use WARMR in our evaluation offers a good foundation from which all of these other methods can be explored in tackling the stated problem as part of future work.

One can think of the Boolean molecular fingerprint and RL representations of the drugs in the stated problem as views in multi-view learning, as both of these representations offer different perspectives in what constitutes the known properties of a drug. In a standard multi-view learning problem the views are typically distinct, meaning that special consideration is made as to the learning algorithm used in building a model for a particular view. Multiple kernel learning [29], which is essentially a form of stacking, has been proposed for such a scenario, where a kernel that is best suited for a particular view is used and the predictions from all views are then combined to form the final prediction. This is in contrast to how we perform our evaluation, because though we considered multiple learning algorithms, a specific learning algorithm is not used for a particular representation.

## Methodology

The LINCS Phase II dataset with accession code GSE70138 provides the expression levels for 978 landmark human genes for 118,050 perturbation conditions. In the metadata, the perturbation conditions are described by their cell line, cell site, drug dosage, drug timepoint, and the applied drug. The Broad Institute identifiers along with their canonical smiles are also provided for the drugs. We were able to map 1,089 of the applied drugs to their DrugBank [36] and ChEMBL [11] identifiers. This is relevant because the SBND knowledge graph uses DrugBank identifiers. The drugs we could map across these databases were applied to only 57,749 of the 118,050 perturbation conditions. For all the aforementioned perturbation condition properties but the drugs, we engineered features for the perturbation conditions using one-hot encoding, and treat them as *base* features. For the propositional representation of the drugs, we converted them into molecular fingerprints using RDKit [19], with 1024 bits, a radius of 4, and useFeatures set to True.

**Fig. 1.**
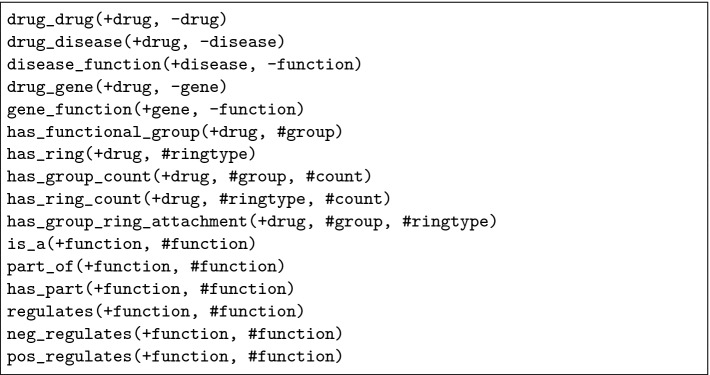
Permitted relations in the body of valid clauses.

For the RL representation of the drugs, we formalised the following relations from the SBND: drug-drug (ChCh-Miner), drug-gene (ChG-Miner), gene-function (GF-Miner), disease-drug (DCh-Miner), and disease-function (DF-Miner). Additionally, we included relationships between functions from Gene Ontology [12], such as is_a and part_of. Furthermore, we included the chemical properties of each drug, such as the presence of rings. In total, this Datalog knowledge base contains 11,175 drugs, 6,869 genes, 45,089 functions and 5,941 diseases. It is available for download at https://github.com/oghenejokpeme/RLCBkb. The hypothesis language we used is given in Fig. 1.

Using this knowledge base, we learned 1,024 frequent patterns using the WARMR algorithm in the Aleph inductive logic programming engine [30] for each drug, which we then use as binary features. WARMR is a levelwise RL algorithm based on a breath-first search of the input knowledge base, as relations are structured as a lattice. It allows for the learning of frequent patterns present in a knowledge base within pre-specified constraints, such as the proportion of the sample space a learned pattern must cover [17]. In Aleph, we used a minimum cover of 5%, a maximum clause length of 20, and 5000 nodes. We should note that there is evidence that suggests that beyond a certain point, increasing the number of learned features leads to no performance gains [26]. With the base features, we then created three data descriptors for the perturbation conditions. One with the base and RL features, another with the base and fingerprint features, and finally, one with the base, RL and fingerprint features. We refer to these datasets as RL, FP, and RL+FP for the remainder of this paper, all of which at this point, have 57,749 samples.

## Evaluation Setup

We used a train-test split in our evaluation and selected only a subset of the samples due to computational complexity. The selection procedure entailed an initial random split of the 57,749 samples into training and testing buckets, 70%-30%. We then randomly selected 7,000 samples from the training bucket for the training set and 3,000 samples from the testing bucket for the test set. This was performed exactly once, and the dataset is available here: http://dx.doi.org/10.17632/8mgyb6dyxv.2. One might argue that this is a paired-input problem, as we are predicting gene expression on pairs of drug perturbation conditions and cancer cell lines. Therefore, we should extend our evaluation to take this into account. We expect that the naive train-test split evaluation approach we have taken will produce more optimistic results than an evaluation procedure for which each entity in a pair present in the test set is also not present in the training set [27]. However, we argue that for the purposes of evaluating standard propositional and RL data descriptors, such an evaluation setting will suffice.

As we mentioned previously, the LINCS dataset contains the gene expression levels of 978 genes. We selected genes that are dissimilar from one another by associated function using Gene Ontology associations at a tree depth of 1, where 0 are the base nodes. This process selected 46 genes, which we used in our experiments. We did this to reduce computational complexity and to select genes that are uncorrelated on a functional level in order to get performance estimates that are generally representative of the complete set of genes. For learning algorithms, we used the least absolute shrinkage and selection operator (LASSO) [34], ridge regression (RR) [14], and random forests (RF) in our evaluation. For LASSO and RR the regularization parameter was chosen using internal 10-fold cross-validation, and the RF models were built with 1000 trees and default settings. The performance metric reported is the coefficient of determination ($R^2$), as we are most interested in the amount of variance explained by the built models. Apart from the standard regression experiments using all three datasets, we also evaluated integrating the predictions made by the RL and FP representations using simple averaging, which is a form of stacking [2]. In this case, we averaged the predictions made using the RL and FP representations. We refer to these results as AVG in the discussion of the evaluation results. All code used for this experiment is available at https://github.com/oghenejokpeme/RLCBexp.

## Results

### Predictive Performance

We observed that on average the RL representation consistently performs worse than all the other representations (see Table 1). For the approaches which combine the RL and FP representations, we found that RL+FP consistently outperforms RL and does not strictly outperform FP on any of the learners. However, RL+FP and FP perform equally well on LASSO. Like RL+FP, AVG also consistently outperforms RL, but is outperformed by FP on both LASSO and RR, but not on RF. These results suggests that the effect the RL representations have when used to augment FP representations depends on two things; the choice of learning algorithm and how the representations are combined. From the mean performance results in Table 1, one might argue that overall, the performance of the representations is generally low. While this is true, we would argue that this is to be expected, as we are attempting to recreate laboratory conditions *in silico*, and predict the expression of 46 genes which often vary in concert and not in isolation of each other. Furthermore, it is worth pointing out that the representations perform reasonably well on some genes, with a maximum $R^2$ of 0.366 when the RL and FP predictions for RF are averaged (Table 1).

**Table 1.** The predictive performance ($R^2$) of the engineered datasets (RL, FP and RL+FP) and the aggregation by mean of the predictions made by RL and FP (AVG) on the learning algorithms. We show the mean with the minimum and maximum performance for the 46 considered genes. The best performing descriptor for each learner is in boldface.

Learner	$R^2$	RL	FP	RL+FP	AVG
LASSO	Mean	0.028	**0.086**	**0.086**	0.081
	min – max	−0.001–0.106	**0.004–0.319**	**0.002–0.320**	0.013–0.271
RR	Mean	0.030	0.090	**0.089**	0.084
	min – max	0.002–0.105	0.009–0.330	**0.008–0.330**	0.013–0.289
RF	Mean	0.068	0.094	0.089	**0.114**
	min – max	−0.012–0.238	−0.047–0.364	−0.050–0.360	**−0.006 – 0.366**

Given the difference in performance between the different methods, we tested for statistical significance using sign tests and paired t-tests. For LASSO, Table 2 shows that RL+FP underperforms when compared to FP, with a ratio of 18–28 of the 46 considered genes and a 0.12% average performance decrease from FP to RL+FP. However, this difference in performance is not statistically significant for both sign test and paired t-test. When AVG is compared to FP, we found that FP performed better on more genes, with a ratio of 17–29. However, we found that when compared to FP, AVG achieves a 9.6% average percentage performance increase over FP, with statistical significance according to the paired t-tests. Further investigation showed that although FP outperformed AVG on more genes, AVG tended to do a lot better than FP on the genes it outperformed FP on, explaining the percentage performance increase. For RR, both RL+FP and AVG both see a statistically significant decrease in average percentage performance when compared to FP. For RF, RL+FP significantly underperforms when compared to FP, but the reverse is true for AVG, with an average percentage performance increase of 178.7%. These results show that for two of the learners we considered, the RL representations can significantly improve predictive performance when used to augment the traditional RL representations. Having established that how the RL and FP representations are combined plays a crucial role in predictive performance, we conjecture that techniques from the multiple kernel learning literature might further improve predictive performance.

**Table 2.** Performance ($R^2$) comparisons between the different datasets for the learning algorithms we considered. The comparisons are structured as approach A/B. For each compared pair, the number of genes for which one strictly outperforms the other is given. Additionally, an asterisk ($*$) and a dagger ($\dagger$) are used to indicate a statistically significant difference in performance with a significance level of 0.05 for a sign test and a paired t-test respectively. Lastly, the average percentage performance increase or decrease when approach A is compared to B is given. It is worth noting that this average percentage performance is calculated by taking the mean percentage difference in performance of genes between A and B, and not simply the percentage difference in mean performance given in Table 1.

Comparison	LASSO	RR	RF
FP/RL	43/3$^{*\dagger }$ (386.5%)	46/0$^{*\dagger }$ (310.9%)	33/13$^{*\dagger }$ (63.7%)
RL+FP/RL	43/3$^{*\dagger }$ (380.9%)	44/2$^{*\dagger }$ (301.5%)	30/16$^{\dagger }$ (26.3%)
RL+FP/FP	18/28 (−0.12%)	13/33$^{*\dagger }$ (−1.4%)	0/46$^{*\dagger }$ (−23.4%)
AVG/RL	46/0$^{*\dagger }$ (355.3%)	46/0$^{*\dagger }$ (276.6%)	46/0$^{*\dagger }$ (191.5%)
AVG/FP	17/29$^{\dagger }$ (9.6%)	14/32$^{*\dagger }$ (−0.07%)	43/3$^{*\dagger }$ (178.7%)
AVG/RL+FP	18/28$^{\dagger }$ (19.7%)	14/32$^{*\dagger }$ (2.0%)	43/3$^{*\dagger }$ (117.6%)

### Explainability

RL enables the introduction of additional background knowledge to the model building process, and it can improve both understandability and predictive performance. In our experiments we were interested in learning frequent patterns present in the knowledge base for the considered drugs. We learned such rules as:
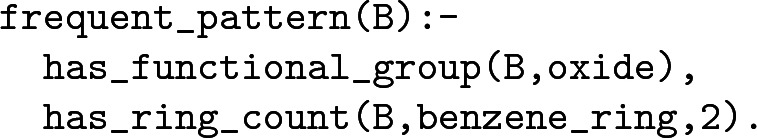


and 
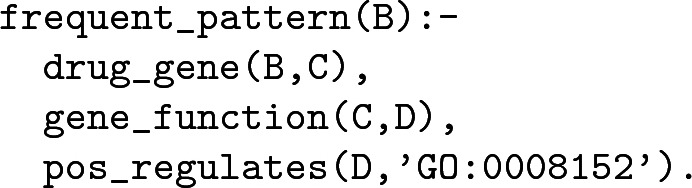


The former rule can be interpreted as *a frequent pattern is for a drug to have an oxide group and two benzene rings.* Note that the standard fingerprint representation of molecules cannot express the simple concept of a molecule having two benzene rings unless a special descriptor ‘two benzene rings’ is pre-generated. Nor can it express the concept of a drug having an oxide group and two benzene rings unless it is pre-generated. To pre-generate all possible descriptors would produce an exponential number of descriptors.

The latter rule can be interpreted as *a frequent pattern is for a drug to target a gene that positively regulates a metabolic process*. Note that is a second-order pattern, the drug targets a gene that in turn regulates metabolism. Most drugs inhibit their targets, and in this pattern the overall result is likely to be decrease in a metabolic process, which is generally desirable in cancer therapy. These examples show that rules are easily understandable by a human reader. One can conjecture that if feature selection is performed when such rules are used as features in a predictive problem, the why of the observed variance in the target could be explained easier. However, it is beyond the scope of this work.

## Discussion

The great success of DNNs is based on their ability *to learn* how to transform a simple input data representation into an effective internal representation. The limitations of the DNN approach are that it requires a large amount of data, the internal representation is obscure, and there is not a general way to encode known problem structure and background knowledge. In many biomedical problems, such as understanding the effect of anti–cancer drugs, it is required to encode a large amount of background knowledge. In this paper we have shown that a hybrid RL approach can *learn* new descriptors that are effective and explainable. The limitations of the hybrid RL approach are that it is a two stage approach rather than end–to–end learning (it is computationally efficient to learn frequent patterns, but they are not necessarily effective), and that the learning model is not differentiable, which makes it more difficult to find model improvements. The main criticism of RL in the past was that it was too inefficient to be applied. However, now, given the vast resources used to train DNNs this no longer applies. It is therefore interesting to consider whether there is a more general way of learning how to improve data representations that combines the advantages of DNNs and the hybrid RL approach, as is the case with deep relational machines [7]. Furthermore, it is worth noting that though the RL representations might be explainable, the interpretability of the models built using them will vary based on the learning algorithm. For example, one might conceivably inspect the important variables of a random forest model, but will find this far more challenging in a deep attention neural network.

## Conclusion

In this paper we report the use of RL representations to enhance the predictive accuracy of traditional propositional data representations for a relevant problem in cancer biology. Apart from improved predictive accuracy, we also learnt explainable patterns underlying common anti–cancer drugs, and built a fully integrated biomedical knowledge base in Datalog which is now publicly available. We intend to investigate other forms of RL as part of future work.
